# Child physical abuse: factors influencing the associations between self-reported exposure and self-reported health problems: a cross-sectional study

**DOI:** 10.1186/s13034-018-0244-1

**Published:** 2018-07-25

**Authors:** Eva-Maria Annerbäck, Carl Göran Svedin, Örjan Dahlström

**Affiliations:** 1 Centre for Clinical Research in Sörmland, Sörmland County Council, Uppsala University, Eskilstuna, Sweden; 2 Child and Adolescent Psychiatry, Department of Clinical and Experimental Medicine, Linköping University, Linköping, Sweden; 3 Barnafrid, Child and Adolescent Psychiatry, Department of Clinical and Experimental Medicine, Linköping University, Linköping, Sweden; 4 Department of Behavioural Sciences and Learning, Linköping University, Linköping, Sweden

**Keywords:** Child physical abuse, Background factors, Perpetrator, Last year exposure, Severity, Frequency, Socioeconomic load, Mental health, Physical health, General health

## Abstract

**Background:**

Child physical abuse (CPA) is an extensive public health problem because of its associations with poor health outcomes. The aim of this study was to examine which of the background factors of CPA committed by a parent or other caregiver relates to self-reported poor health among girls and boys (13; 15 and 17 years old): perpetrator, last year exposure; severity and frequency; socioeconomic load and foreign background.

**Methods:**

In a cross-sectional study in a Swedish county (n = 8024) a path analysis was performed to evaluate a model where all background variables were put as predictors of three health-status variables: mental; physical and general health problems. In a second step a log linear analysis was performed to examine how the distribution over the health-status categories was different for different combinations of background factors.

**Results:**

Children exposed to CPA reported poor health to a much higher extent than those who were not exposed. In the path analysis it was found that frequency and severity of abuse (boys only) and having experienced CPA during the last year, was significantly associated with poor health as well as socioeconomic load in the families. Foreign background was significantly negatively associated with all three health indicators especially for girls. Neither mother nor father as perpetrator remained significant in the path analysis, while the results from the log linear analyses showed that mother-abuse did in fact relate to poor general health and mental as well as physical health problems among boys and girls. Father-abuse was associated with poor mental health if severe abuse was reported. Poor mental health was also associated with mild father-abuse if exposure during the last year was reported.

**Conclusion:**

Despite the limitations that cross-sectional studies imply, this study provides new knowledge about factors associated with poor health among physically abused children. It describes details of CPA that have significant associations to different aspects of poor health and thus what needs to be addressed by professionals within mental health providers and social services. Understanding how different factors may contribute to different health outcomes for exposed children is important in future research and needs further studies.

## Background

### Definitions

#### Child physical abuse (CPA)

Physical violence against a child executed by a parent or a caregiver.

#### Caregiver

A person who had parental responsibility for the child at the time of the abuse.

#### Child

A person younger than 18 years.

CPA is an extensive public health problem because of its high prevalence and its associations with adverse health outcomes [1, 2]. There is a great amount of research showing that there are strong enduring effects of physical abuse and other adverse childhood experiences on mental and/or physical health in adulthood [3–7]. In a previous study it was found that CPA was associated with health problems among boys and girls and that the associations were stronger among the children who reported repeated CPA [8]. The impact of child abuse on health cannot be explained by any single cause since health depends on a complex web of different factors [9]. Kiser et al. [10] emphasize that research is needed about the mechanisms of the traumatic experiences. They mention, for example, type of trauma, age of exposure, duration, frequency, severity, and the relationship to the perpetrator as examples of such details identified in the literature that promote a nuanced picture of CPA [10].

### Perpetrator patterns

*Betrayal trauma* or trauma perpetrated by someone with whom a victim is close has been shown to be associated with young adults’ physical and mental health difficulties to a greater extent than other forms of trauma [11]. This is in good agreement with *Attachment theory*, that provides a universal explanation of implications of CPA and points to the difference between being exposed to violence by parents and to violence committed by other adults. If the person who should represent *the Secure Base* for a child is the same person who hurts the child, this seriously harms the vital relationship between child and parent and over time the health of the child [12–15].

In general, few studies have examined the relationship between health problems among physically abused children and gender of the perpetrator. Already, in 1993, Allen and Epperson [16] pointed out the lack of research on gender differences among the perpetrators of child maltreatment and argued that a differentiated knowledge would result in improved understanding of, among other things, the consequences of child abuse [16]. They proposed that there might have been different reasons for this lack of research such as (1) “a males-only perspective”; (2) “the mother-blaming perspective” or (3) the choice of study group, which all imply limitations. They considered that studies of registered cases distort results because men, for example, are overrepresented as perpetrators in police statistics (Allen and Epperson [16], p. 545–50). In a study of youth victimization in the U.S. [17], it was found that males were overrepresented as perpetrators and boys as victims in physical abuse by caregivers and that “Many violence types were more severe when perpetrated by males versus females as indicated by higher injury rates and greater victim fear” (Hamby et al. [17], p. 915). In a Swedish study from 2008 there were almost as many women as men among the perpetrators of CPA, even though there was a greater percentage of males who had exposed the children to repeated violence [18]. In a recent Swedish study, no differences in health outcomes were found whether the mother or the father was the perpetrator of the abuse [19].

### Time point for the abuse/last year exposure

Previous studies have found an increase of reports of physical abuse with age [20, 21]. In a study conducted in 2008, it was found that 13 year olds reported 12.1% “lifetime experience” of CPA; 15 year olds 18.6% and 17 year olds 16% [18]. The increase with age is important to examine further since there could be different explanations of this. The question is, if there really is an increase of exposure to CPA among teenagers or if these figures depend on different reporting patterns in different age groups? In the current study a question about experience of CPA during the last year thus was added in order to be able to test how this might influence associations with health-factors.

### Frequency and type/severity of abuse

In a previous study it was found that there was a dose–response effect between frequency of CPA and self-reported ill health [8]. In Sweden where all corporal punishment has been considered a crime for almost 40 years, the use of physical violence in child rearing has become more unusual.

The immediate consequences of CPA are physical pain, acute stress and potential physical injuries. The most common injuries from physical abuse are marks from beatings and kicks. Bruises in unusual places or bruises of different ages might indicate abuse. But CPA also includes more severe violence and injuries which can cause life-long consequences or even be life-threatening [22, 23]. In a Swedish school survey in 2011 (15–16 year olds), one-third of the children who reported CPA (in total 13.8%), reported that they had been exposed at some point to more severe types such as harder beatings with the hand/fist, kicked, scalded, squeezed on the throat or that they had been beaten with an object [24]. In a study of cases of CPA reported to the police in Sweden, the share of severe cases including striking the child with an object or against a surface, choking the child or beating up the child was 41% [25]. There are reasons to believe that the more severe forms have greater impact, since they are likely to be more painful, more frightening and thereby also more psychologically traumatizing. To the best of our knowledge, there are no studies on how different types/different severity of CPA influences the relations with poor self-reported health.

### Socioeconomic load and foreign origin

Social and economic factors are seen to have great impact on health among youths as well as among adults. Social and economic inequality predicts health problems such as high body-mass index, psychological and physical problems as well as social problems among adolescents and is therefore an important factor to consider when studying poor health among youths [26, 27]. According to studies of child poverty in Sweden carried out by Save the Children, the groups subjected to the strongest effects are immigrant families and single-parent families [28]. Children with foreign origin, meaning that both parents are born abroad have been seen to have an increased risk of being exposed to CPA in Sweden [18, 24]. Widom et al. [7] discussed whether consequences of abuse differ for children of different racial and ethnic backgrounds. They describe varying and partly opposing theories: (1) the racial inference theory which predicts that effects of abuse would be about the same independent of origin, (2) the double jeopardy theory implying stronger associations with poor health for children of minority status and exposure for abuse, and (3) the theory of resilience which states that the effects are less for children of other origins due to the fact that they have grown up with other stressors in life and other cultural factors that can buffer the effects of abuse [7].

In summary, the above presented literature review shows that there is limited knowledge on how different factors interact with each other and how these contribute to poor health among children exposed to CPA. The current study aims to examine four different categories of such factors that have been seen to have potential influence: perpetrators, severity, frequency and time point of the abuse.

## Methods

This study aims to investigate potential factors by which CPA perpetrated by caregivers might be associated with self-reported poor health. We hypothesized that (1) parental physical abuse; (2) severity and frequency of CPA and (3) time point—exposure to CPA within the last year, negatively influence the health of children exposed to CPA. More specifically, the first aim was to examine which of the factors: relation to the perpetrator (mother, father, stepparent), last year exposure, type of abuse, frequency of abuse, socioeconomic load and foreign origin, relates to poor self-reported general health, physical health and/or mental health problems among girls and boys exposed to CPA. The second aim was to examine if, and if so in what way, background factors such as mother-abuse, father-abuse (both with stepparent-abuse as baseline), gender, last-year exposure, socio-economic load and foreign origin are associated with health-status (poor self-reported general health, physical health problems and mental health problems).

### Data collection

All pupils in grade seven and nine in compulsory school and grade two in upper secondary school (13, 15 and 17 years old) in Södermanland County, Sweden, were invited to participate in a population-based study in 2011 (n = 9600). The Centre for Public Health conducted the study in collaboration with the Centre for Clinical Research at Södermanland county council. School employees managed questionnaire distribution and collection. The questionnaires were completed in classrooms during school hours. All answers were anonymous and were returned in sealed envelopes. The children were informed orally and in writing about the purpose of the study, and that they could discontinue or refuse to participate in the study. They were also told that the collected information would remain confidential. The schools informed parents of pupils in grade seven about the survey and that they could prevent their children from participating by informing the school about this. The parents of pupils in grade nine and grade two were not informed since children > 15 years of age in Sweden are considered to have the right to make their own decisions in such matters.

### Study sample

**Fig. 1:**
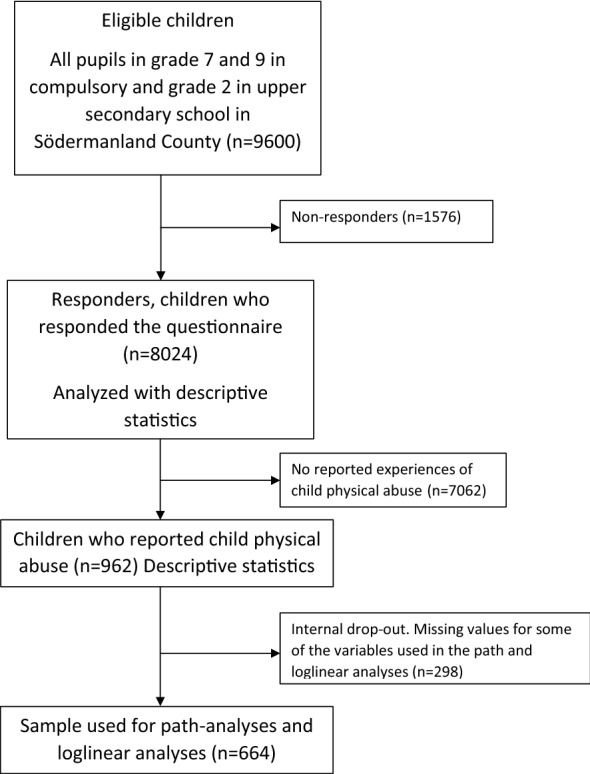
Flow chart showing eligible children and study groups

Response rates were 86% in grade seven (13 years old), 84% in grade nine (15 years old) and 77% in grade two (17 years old). The drop-outs consisted mainly of children absent from school on the days the survey was given out. These children were probably absent because of illness or truancy. A second chance was given to the non-respondents. The final sample consisted of 8024 respondents. The internal data loss on individual questions used in this study was less than 2% apart from parental employment, which was 9%. The total numbers of individuals included in different analyses vary because of internal dropout for some of the questions. For further information on children included in different analysis see flow chart (Fig. 1).

### The questionnaires

The main purpose of the survey was to collect data on young people’s health and the children were asked about health, lifestyle and life experiences. The same kind of survey had been conducted previously on three occasions. Material from the survey in 2008 has been used in previous studies on CPA [8, 18]. This paper focuses on CPA and related questions from the 2011 survey [29], which was conducted in a new sample. In the 2011 questionnaire two new questions were added. The first new question was about the type/severity of CPA with answer options in a modified version of Conflict Tactic Scale (CTS), Parent–Child Version. CTS is an instrument for identifying child abuse and distinguishes physical abuse in two subscales; Corporal punishment (mild abuse) and Severe Physical Assault [30]. The second new question was whether abuse occurred during the last year or not. The questions had multiple answer options except for the question about time point for the abuse, which had two answer options (Table 1).

**Table 1:** Child physical abuse variables from the questionnaire Liv & Hälsa ung 2011 (Life & Health Young) and eligible answer options

Item	Answer options	Coded responses^a^
Have you been slapped on the ear/been beaten by an adult?	No Yes, once Yes, several times	No Yes Yes
How often and by whom have you been slapped on the ear/been beaten?^a,c^	Never, by mother, father, mothers partner, fathers partner, another adult Yes, once or twice by mother, father, mothers partner, fathers partner, another adult Yes, several times by mother, father, mothers partner, fathers partner, another adult	*Frequency* was coded as low if the child answered “once or twice” and high if the child answered more than twice *Perpetrator* was coded mother, father, stepparent. If the child only answered “another adult” answers were excluded
In what way have you been beaten by an adult?^b^	Been slapped on the ear, been shaken, pulled by the hair Lighter beatings with the hand/fist Harder beatings with the hand/fist Beaten with a stick or a belt Another way	*Severe abuse* was coded if the child answered that he or she had been exposed to harder beatings with hand/fist and/or had been beaten with an object
Have you told anyone that you have been slapped on the ear/been beaten?^b^	Yes, told siblings, peers, girlfriend or boyfriend Yes, told parent/other close adult Yes, told school staff, social services, police, health care personnel or similar No	*Not told any authority* was indicated if the child did not choose this answer option
Has this (that you have been slapped on the ear/been beaten) occurred during the last 12 months?	Yes No	*Last year* exposure was codes if the child answered yes

^a^Dichotomized coded items

^b^Response options are multiple choice

^c^Matrix question with 15 answer options

### Measures

#### CPA

Variables of CPA are described in Table 1.

#### Health indicators

*Poor general health* was designated when the child answered “bad” or “very bad” to the question “How is your health in general?”. *Physical health problems* were indicated if the child answered “Yes, almost every day” to at least one of the alternatives in the question “How often during the last 3 months have you had the following complaints: headache, migraine, stomach-ache (not menstrual pain), ringing in the ears/tinnitus, and pain in back/hips/shoulders?” *Mental health problems* were indicated if the child answered “Yes, almost every day” to at least one of the alternatives in the question “How often during the last 3 months have you had the following complaints: insomnia, anxiety and worry, depression?”

#### Background indicators

*Socioeconomic load* was measured by two questions. “What is your mother/father doing?” (with answer options: working, studying, unemployed, on sick leave, other) and “How do you live?” (with answer options of different types of accommodations: rented apartment, condominium, own townhouse or villa which defined the question). *Socioeconomic load* was designated if the child reported that one or both parents were unemployed/on sick leave and that the family lived in rented accommodation. (In Sweden, those who live in rented accommodation have lower average incomes than those who own their home [31].

*Origin* was dichotomized as (1) At least one parent born in Sweden (*Swedish origin*) (2) Both parents born abroad (*Foreign origin*).

### Statistical analyses

Descriptive statistics were calculated using standard methods: frequencies and cross-tabulations.

The first aim, to investigate potential factors by which CPA perpetrated by caregivers might be associated with self-reported poor health, was examined by path analysis starting with a model where all background variables—frequency of abuse, severity of abuse, last-year exposure, socioeconomic load and foreign origin—were put as predictors of each health-status variable—poor general health, physical health problems, and mental health problems. Thereafter a stepwise procedure was conducted where the least significant path was removed, until only significant (*p *< 0.05) predictors remained. The final model represents the theoretical model, where background variables are assumed to cause health status, which best fits with the data. This was done separately for all participants, for girls, and for boys. To take account of the categorical character of data, the models were estimated using the mean and variance adjusted weighted least squares (WLSMV) estimator in the Mplus statistical modeling program. The model was evaluated using several different fit indices [32] provided by the Mplus output: Chi square statistics, Root Mean Square Error of Approximation (RMSEA), Comparative Fit Index (CFI), Tucker-Lewis Index [TLI, also known as the Non-normed fit index (NNFI)] and the Weighted Root Mean Square Residual (WRMR). The model was judged as having good fit when the overall picture of fit indices indicated good fit and excellent if all of them indicated good fit: RMSEA ≤ 0.05, CFI and TLI ≥ 0.95, and WRMR < 0.90 (see e.g. [33]).

The second aim, to examine if, and if so in what way, health-status (poor self-reported general health, physical health problems, mental health problems) are associated with abuse [mother-abuse and father-abuse (both with stepparent-abuse as baseline)] and in possible interactions with gender, last-year exposure, socioeconomic load and foreign origin, was examined by log linear analysis and Chi square tests of homogeneity. Combinations of variables included:

- at least one of the health status variables—poor general health, physical health problems, mental health problems.
- any of mother-abuse, father-abuse, gender, last-year exposure, socioeconomic load or foreign origin.

The procedure tests the highest-order interaction and if non-significant, it is excluded. Thereafter, the next highest-order interactions are tested, and so on. In case of a significant interaction a split of the data is made based on one of the variables and the interactions among the remaining variables are tested for in the split datasets. 2-way interactions were examined by Chi square statistics using Cramer’s V as a measure of effect size and using standardized residuals less than − 2 (indicating unexpectedly low frequencies) or larger than 2 (indicating unexpectedly high frequencies) to describe what cells (combination of variable values) explain the significance. Analyses required expected frequencies ≥ 1 for all cells and < 5 for at most 20% of the included cells. Therefore 5-way interactions were tested, followed by all lower-level interactions that were not already included in any higher-order interaction that fulfilled the required criteria.

The path analysis was performed using Mplus Version 7.4 [34] and the log linear analysis was performed using IBM Statistical Package for the Social Sciences (SPSS) version 22.0.

## Results

The results show that 962 (12.0%) of the 8024 children reported that they had been exposed to CPA committed by a parent or other caregiver and that 30% of these reported that they had been abused during the last year. Perpetrators were usually biological parents (92.6%) while stepparents accounted for 7.4% of perpetrators. Descriptives of CPA variables within the total group are presented in Table 2. Eight percent of all exposed children (n = 962) had told an authority (school personnel, social services, police and similar) about the abuse. Mental health problems were reported by 11.3% of the not exposed (n = 7062) and of 31.6% of the CPA group (p < 0.001). Physical health problems were reported by 10.9% of the not exposed and 22.5% of the CPA group (p < 0.001). Poor general health was reported by 2.3% of the children not exposed to CPA compared with 10.5% among the exposed (p < 0.001). In the total study sample, the children of foreign origin reported CPA more often (19.0%) than children with Swedish origin (11.0%), Children of foreign origin reported mental health problems more often (16.6%) than those with Swedish origin (13.8%), Physical poor health was reported less often by children of foreign origin (11.0%) than of those with Swedish origin (13.8%) and poor general health to about the same extent in both groups.

**Table 2:** Description of child physical abuse among those abused by caregivers presented as numbers and percentages of characteristics within the exposed group in parentheses

	n	Perpetrator(s)						
		Mother (but not father)	Father (but not mother)	Mother and father	Stepparent (but not biological parent)	Last year exposure	Type of abuse: severe	Frequency: more than twice
Grade								
Compulsory school, grade 7								
n = 2485*	228	71 (31.1)	85 (37.3)	56 (24.6)	16 (7.0)	107 (46.9)	49 (21.5)	75 (32.9)
Compulsory school, grade 9								
n = 2762*	351	105 (29.9)	125 (35.6)	92 (26.2)	29 (8.3)	117 (33.3)	63 (17.9)	128 (36.5)
Upper secondary school, grade 2								
n = 2777*	383	114 (29.8)	139 (36.3)	104 (27.2)	26 (6.8)	89 (23.2)	76 (19.8)	137 (35.8)
Gender								
Boys								
n = 4057*	430	97 (22.6)	181 (42.1)	124 (28.8)	28 (6.5)	127 (29.5)	83 (19.3)	155 (36.0)
Girls								
n = 3935*	524	191 (36.5)	164 (31.3)	126 (24.0)	43 (8.2)	183 (34.9)	104 (19.8)	184 (35.1)
Parents foreign born								
≥ 1 parent born in Sweden								
n = 6555*	688	220 (32.0)	262 (38.1)	148 (21.5)	58 (8.4)	227 (33.0)	124 (18.0)	224 (32.6)
Both parents born abroad								
n = 1217*	228	56 (24.6)	69 (30.3)	95 (41.7)	8 (3.5)	68 (29.8)	51 (22.4)	97 (42.5)
Socio-economic load								
No								
n = 7419*	840	260 (31.0)	304 (36.2)	213 (25.4)	63 (7.5)	263 (31.3)	151 (18.0)	281 (33.5)
Yes								
n = 530*	105	26 (24.8)	40 (38.1)	33 (31.4)	6 (5.7)	44 (41.9)	34 (32.4)	51 (48.6)
Total								
n = 8024*	962	290 (30.1)	349 (36.3)	252 (26.2)	71 (7.4)	313 (32.5)	188 (19.5)	340 (35.3)

* Based on the total study sample

In the path and loglinear analyses cases with missing values were excluded, resulting in a slightly smaller sample (n = 664). Drop-outs (n = 298) reported slightly more exposure (examined by cross-tabulations), meaning that, if anything, associations and relations from these analyses are slightly under-estimated.

### Health status with different background variables

**Fig. 2:**
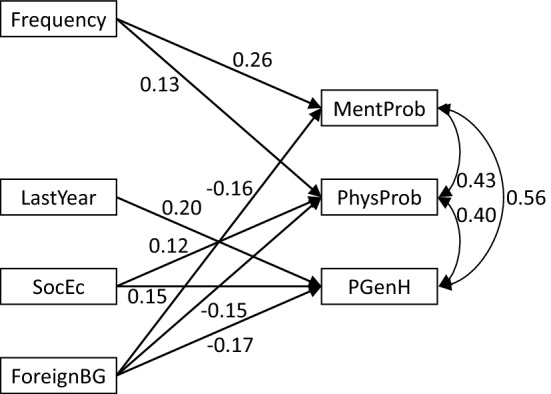
Girls and boys; path analysis for the association between background variables and health problems. NB all coefficients are standardized and significant at p < 0.05. MentProb = mental health problems (0 = no, 1 = yes); PhysProb = physiological health problems (0 = no, 1 = yes); PGenH = poor general health (0 = no, 1 = yes); Frequency = frequency of abuse; LastYear = last year exposure (0 = no, 1 = yes); SocEc = socio-economic load (0 = no, 1 = yes); ForeignBG = foreign background (0 = no, 1 = yes)

**Fig. 3:**
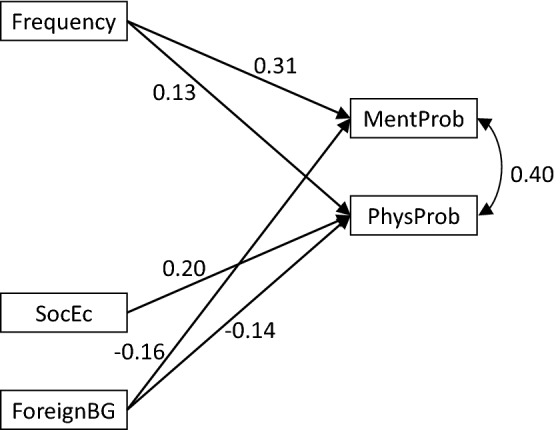
Girls; path analysis for the association between background variables and health problems. NB all coefficients are standardized and significant at p < 0.05. MentProb = mental health problems (0 = no, 1 = yes); PhysProb = physiological health problems (0 = no, 1 = yes); Frequency = frequency of abuse; SocEc = socio-economic load (0 = no, 1 = yes); ForeignBG = foreign background (0 = no, 1 = yes)

**Fig. 4:**
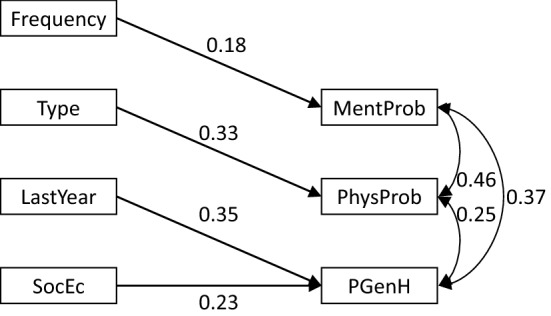
Boys; path analysis for the association between background variables and health problems. NB all coefficients are standardized and significant at p < 0.05. MentProb = mental health problems (0 = no, 1 = yes); PhysProb = physiological health problems (0 = no, 1 = yes); PGenH = poor general health (0 = no, 1 = yes); Frequency = frequency of abuse (0 = not more than twice, 1 = more than twice); Type = type of abuse (0 = minor, 1 = severe); LastYear = last year exposure (0 = no, 1 = yes); SocEc = socio-economic load (0 = no, 1 = yes)

Stepwise deletion of non-significant variables—mother-abuse, father-abuse (stepparent as baseline), last-year exposure; and the other background variables—resulted in different models with good fit for all, for boys and for girls (Figs. 2, 3, 4). Notably, perpetrator (mother-abuse and/or father-abuse, with stepparent-abuse as baseline) did not remain significant in any of the models, while last year exposure showed a significant association with poor general health, mainly for boys.

*Mental health problems* were related with higher frequency of abuse, for boys as well as for girls (Figs. 2, 3, 4).

*Physical health problems* were associated with higher frequency of abuse, with socioeconomic load and negatively with foreign origin (Fig. 2). These associations were also present when girls were examined separately (Fig. 3). For boys there was a significant association with severe type of abuse (Fig. 4).

*Poor general health* was associated with last year exposure, socioeconomic load and negatively with foreign origin (Fig. 2), although origin did not remain significant for boys (Fig. 4) and there was no significant relation with poor general health in the gender-specific analyses (Figs. 3, 4).

The pairwise associations between the variables are presented in Table 3.

**Table 3:** Associations among variables shown by Pearson correlation (Phi coefficients, since variables are all binary)

	1	2	3	4	5	6	7	8	9	10
Health status										
1. Mental health problems										
2. Physical problems	0.30***									
3. General health	0.33***	0.23***								
Predictors										
4. Mother-abuse	0.05	0.02	0.05							
5. Father-abuse	0.00	0.03	0.00	− 0.41***						
6. Last-year exposure	0.10**	0.11**	0.14***	0.12**	0.01					
7. Gender/girls	0.14***	0.20***	0.11**	0.12**	− 0.11**	0.06				
8. Frequency	0.21***	0.13**	0.10*	0.08*	0.12**	0.21***	− 0.02			
9. Type	0.11**	0.12**	0.12**	0.00	0.04	0.21***	− 0.01	0.40***		
10. Socioeconomic load	0.04	0.09*	0.09*	0.01	0.05	0.04	0.02	0.13***	0.08*	
11. Foreign background	− 0.10*	− 0.08*	− 0.08*	0.06	0.11**	− 0.04	− 0.04	0.04	0.02	0.22***

* p < 0.05

** p < 0.01

*** p < 0.001

### Health status and associations with background variables

The higher-order interactions (3-way and higher) are presented and explored in Table 4.

**Table 4:** Adolescents with experience of care-giver abuse and how their general, mental and physical health problems are distributed over different background variables (only significant interactions, 3-way and higher, in *italics*, are presented)

Interaction	*p*	OR (95% CI)
Mental health problems		
Mental*mother-abuse*LYexp*foreign	0.011	
Foreign = no		
Mental*mother-abuse*LYexp	0.041	
LYexp = no		
Mental*mother-*abuse*	0.043	1.63 (1.01–2.63)
LYexp = yes		
Mental*mother-*abuse*	0.294^α^	0.71 (0.37–1.35)
Foreign = yes		
Mental*mother-abuse*LYexp	0.043	
LYexp = no		
Mental*mother-*abuse*	0.100^β^	0.45 (0.17–1.18)
LYexp = yes		
Mental*mother-*abuse*	0.636^β^	1.52 (0.26–8.77)
Mental*father-abuse*LYexp*type	0.042	
Type = mild		
Mental*father-abuse*LYExp	0.003	
LYexp = no		
Mental* father-*abuse*	0.040	0.63 (0.40–0.98)
LYexp = yes		
Mental* father-*abuse*	0.027	2.27 (1.09–4.73)
Type = severe		
Mental*father-abuse* LYExp	0.494	
LYexp = yes or no		
Mental*father-*abuse*	0.559^α^	0.89 (0.63–1.30)
Physical health problems		
Physical *type*gender	< 0.001	
Boys		
Physical*type	< 0.001	5.54 (2.68–11.45)
Girls		
Physical*type	0.593^α^	1.16 (0.67–2.00)
Physical*mother-abuse*LYexp	< 0.001	
LYexp = no		
Physical*mother-*abuse*	0.032	1.67 (1.04–2.67)
LYexp = yes		
Physical*mother-*abuse*	0.009	0.44 (0.24–0.82)
General health problems		
General *mother-abuse*LYexp	0.035	
LYexp = no		
General*mother-*abuse*	0.033	2.25 (1.05–4.81)
LYexp = yes		
General*mother-*abuse*	0.384	0.72 (0.34–1.52)
Mental and physical health problems		
Mental*physical*mother-abuse*gender	0.024	
Boys		
Mental*physical*mother-*abuse*	0.177^γ^	
Mental*physical	< 0.001	4.51 (2.22–9.19)
Girls		
Mental*physical*mother-*abuse*	0.038	
Mother-abuse (no) mental*physical	0.055	2.01 (0.98–4.12)
Mother-abuse (yes) mental*physical	< 0.001	5.40 (2.96–8.96)

Odds ratios (OR) show the increased odds of health problem in case of presence of the other variable included compared to not

Variables: general = general health problems (no, yes), mental = mental health problems (no, yes), physical = physical health problems (no, yes), Genmoo der (boys, girls), mother-abuse (no, yes), father-abuse (no, yes), LYexp = last-year experience (no, yes), type = type of abuse (mild, severe), foreign = foreign background (no, yes)

*p*-values for three- or four-way interactions are based on Likelihood Ratios (which tests the null hypothesis that the interaction is zero) and *p*-values for two-way interactions, in order to explain higher-order interactions, are based on Chi square tests of homogeneity. The strength of (significant) two-way interactions are given by odds ratios (OR). (e.g. mental*physical has OR = 4.51 means that adolescents with experience of physical health problems have 4.51 times higher odds to also have mental health problems compared to adolescents without physical health problems.)

^α^Non-significant test is given as reference to explain overlying three-way interaction (i.e. that the two-way interaction in this subgroup is different compared to the other subgroup(s) included in the three-way interaction)

^β^The two-way interactions are not significant, but they have different directions (i.e. odds ratios are on opposite sides of 1), which explains the significant three-way interaction

^*γ*^Non-significant test is given as reference to explain overlying four-way interaction (i.e. that the three-way interaction in this subgroup is different compared to the other subgroup included in the four-way interaction)

#### Mental health problems

Mental health problems were differently distributed over mother-abuse and last-year exposure for those with and for those without foreign origin p = 0.011. For those with no foreign origin the distribution of reported mental health problems depending on reported mother-abuse differed between those with and those without last-year experience, p = 0.041. Those (with no foreign origin) without last-year exposure had higher odds (OR = 1.63; 95% CI 1.01, 2.63) of mental health problems if reporting mother-abuse, p = 0.043. For those (with no foreign origin) with last-year experience there was lower odds (OR = 0.71; 95% CI 0.37, 1.35) of mental health problems if reporting mother-abuse (although not significant). For those with foreign origin, the distribution of reported mental health problems depending on reported mother-abuse also differed between those with and those without last-year experience, p = 0.043, but in the opposite direction compared to those with no foreign origin. Among those (with foreign origin) without last-year experience, there was lower odds (OR = 0.45; 95% CI 0.17, 1.18) of mental health problems if reporting mother-abuse, and although non-significant this was different from those (still with foreign origin) with last-year experience where there was higher odds (OR = 1.52; 95% CI 0.26, 8.77) of mental health problems if reporting mother-abuse (also non-significant).

Mental health problems were also related to father-abuse; mental health problems were differently distributed over father-abuse and last-year exposure for those with experience of mild and for those with experience of severe abuse, p = 0.042. For those experiencing mild abuse, the distribution of mental health problems depending on father-abuse differed between those reporting last-year experience and those who did not, p = 0.003. For those (experiencing mild abuse) without last-year experience there was a lower odds (OR = 0.63; 95% CI 0.40, 0.98) of mental health problems if reporting father-abuse, p = 0.040, while those (experiencing mild abuse) with no last-year experience showed a higher odds (OR = 2.27; 95% CI 1.09, 4.73) of mental health problems if reporting father-abuse, p = 0.027. For those experiencing severe abuse these distributions were not significant, p = 0.494, nor were there any differences in distributions of mental health problems depending on last-year-experience (for those who experienced severe abuse), p = 0.559.

#### Physical health problems

Physical health problems were differently distributed over type of abuse for boys and girls, p < 0.001. For boys there was a significant association between physical health problems and type of abuse (OR = 5.54; 95% CI 2.68, 11.45), i.e. more physical health problems with severe abuse, p < 0.001, while there was no such significant association for girls (OR = 1.16; 95% CI 0.67, 2.00).

The distribution of physical health problems was differently distributed over mother-abuse for those without, compared to those with, last-year experience, p < 0.001. In cases of no last-year experience mother-abuse showed a significant association with physical health problems (OR = 1.67; 95% CI 1.04, 2.67), p = 0.032, but in cases of no last-year experience, the association was in the opposite direction (OR = 0.44; 95% CI 0.24, 0.82), p = 0.009.

#### General health problems

Distribution of general health problems was differently distributed over those without and those with reporting mother-abuse for those with last-year exposure compared to those with no last-year exposure, p = 0.035. In cases with no last-year experience there was a significant association between general health problems and mother-abuse (OR = 2.25; 95% CI 1.05, 4.81), p = 0.033, indicating higher odds of general health problems if experiencing mother-abuse, but in cases of last-year exposure, there was no such significant association (OR = 0.72; 95% CI 0.34, 1.52).

#### Mental health and physical health problems

Distribution of mental health problems, physical health problems and mother-abuse were different for boys and girls, p = 0.024. For boys, the association between mental and physical health problems were not significantly different between boys not experiencing and boys experiencing mother-abuse, p = 0.177, but there were nevertheless a significant association between mental health problems and Physical health problems (OR = 4.51; 95% CI 2.22, 1.52) indicating higher odds of mental health problems for boys reporting physical health problems, p < 0.001. For girls, the associations were similar, but unlike boys the three-way interaction between mental health problems, physical health problems and mother-abuse was significant, p = 0.038. There was a positive association between mental and physical health problems for girls with no experience of mother-abuse (OR = 2.01; 95% CI 0.98, 4.12), although non-significant, but that association was significantly stronger for girls being abused by the mother (OR = 5.40; 95% CI 2.96, 8.96), p < 0.001.

## Discussion

The present study aimed to contribute to the field of research on CPA by examining how different characteristics of abuse were associated with poor health in a group of children who reported that they had been exposed to physical abuse by a caregiver. The study shows that children exposed to CPA reported poor health to a higher extent than those who were not exposed. Associations between characteristics of the abuse and other background factors and poor health were examined in two different types of analysis: one path analysis and in addition in log linear analysis.

The hypothesis, that violence perpetrated by mothers and fathers (with stepparent as base-line), is associated with the worst outcomes of CPA was not supported by the results in the path analysis where all the prerequisite variables were put together in a base-model. Since the hypothesis might still be valid in groups with different characteristics of background variables, this issue was examined further. The results from the log linear analyses showed that mother-abuse did in fact relate to mental as well as physical and general health problems. For those experiencing mild father-abuse there was a positive association with mental health problems if the abuse had occurred during the last year otherwise there was a negative association. Mother-abuse is associated with poor self-reported health more often than father-abuse and does not seem to be affected by last year experience in the same way. Why CPA performed by mothers has more effect on health problems might be explained by attachment theory since mothers are often the most important attachment figures [12, 15]. This is partly supported by the study of Nilsson et al. [19] where children abused by their mothers reported their mothers’ parenting as more negative when mothers only or both parents were perpetrators of the abuse compared to only fathers as perpetrators. Further studies are required, to more clearly elucidate the question of the impact of CPA performed by primary attachment figures in comparison with violence from other caregivers. Previous research has given conflicting results on this point [10, 11].

Further, in the path analysis it was found that frequency and severity (boys only) of abuse and having experienced CPA during the last year (especially boys) was significantly associated with poor health as well as socioeconomic load in the families. The fact that higher frequency of abuse and socioeconomic problems are strongly associated with self-reported ill health among boys and girls is consistent with other studies [8, 27].

Another demographic factor, foreign origin had a partial opposite influence on self-reported health. The exposed children with foreign origin reported significantly fewer health problems than the exposed children of Swedish origin, although the association was not significant in the separate analysis for boys. One possible explanation of this difference might be cultural differences connected to CPA. In Sweden, where all violence against children has been banned for almost 40 years, exposure to violence from a caregiver has come to be viewed as a deviant experience. For the Swedish children this experience may be perceived as exclusion in society and lead to marginalization for the abused children and thus result in poorer health [35, 36]. The same behavior might not have the same impact if the internal family values are more permissive towards corporal punishment and is normalized in families or groups of children of foreign origin where corporal punishment is more prevalent [18, 24]. These results thus seem to support the hypothesis of resilience in more disadvantaged groups due to the probability that these children have grown up with other stressors in life and other cultural factors that can buffer the effects of abuse, as described in Widom et al. [7]. Another assumption is that CPA is not associated with other family problems to the same extent in families of foreign origin as in families with Swedish origin, where all violence against children is considered to be abnormal and prohibited. Perhaps families of foreign origin can offer their children support in a way that is protective against poor health despite the violence.

Finally, the study shows that a relatively high proportion of older children in their teens report being abused during the last year, especially boys and that this contributes to poor self-perceived health. The finding of high proportion of last year experience of CPA corresponds with Finkelhor et al. who found that last year experience of abuse was frequently reported by 14–17 years old adolescents [20]. In the current study all the three age groups (13, 15 and 17 years old) reported that they had been abused in the last year, although the prevalence of last year exposure was more common at the younger ages. These results correspond with Radford et al. who found that last year experiences rise from ages 11–12, peak between ages 13–16 and then decline [21].

### Limitations/methodological considerations

The present study has several possible limitations. First, the cross-sectional design implies lack of temporal ordering of incidents, which limits the possibility of addressing the question of causality [37]. Second, the data are based on self-reports of experiences in the past. Gilbert et al. [1] discussed the complexity of the phenomenon of child abuse and of research design in the field. They proposed that it would be desirable that research on consequences of child abuse consisted of prospective cohort studies, but in the same article they also discussed the problems such designs imply since official cases of abuse do not represent the population of all abused children. The retrospective nature of data also implies potential recall bias since children cannot report occasions that happened during their first years. In addition to this, another limitation of the study might be that we do not have data on the actual age of exposure, except if it occurred in the last year. Age of exposure might affect the outcome in health [10]. Third, this study focuses solely on physical abuse which might be another limitation since there are not adjustments for concurrent other types of abuse. Child abuse, however, is a complex phenomenon and is characterized by multifactorial patterns and it is a challenge to find adequate methods in this research-area. There are also difficulties in determining causality between the abuse experience and health problems in longitudinal studies because there are several unknown factors that might affect the outcomes. In a Swedish study of CPA-cases reported to the police, a 5-year follow-up revealed that much had happened in the children’s lives after the reports that were not directly linked to the abuse [38]. In addition, there are several other changes that occur naturally in young people’s lives. Overall, these circumstances may affect the health of young people as well as experiences of abuse do and could be regarded as a mutual limitation in different types of study design [39]. Fourth, another type of bias, dependent measurement bias, implies that false associations can occur due to problems in measurement [40, 41]. One source of such bias in connection to questionnaire based studies, is that the stable personality traits of the participants in a study means that they tend to consistently report the most “negative” alternatives while others score the most “positive” [41]. In this study however, it might be assumed that pupils would rather tend to overreport, alternatively underreport both exposure (abuse) and outcome (health) and that skewness in results would not arise. This also touches upon the problem of that outcome and predictor variables were assessed by the same individual, which tends to strengthen relationships [42]. It was on the other hand not in the framework for this large study where data was collected anonymously from the children themselves. Further the use of different informants bring new problems with informant variance (see e.g. Edelbrock [43]) concerning mental health problems [44] and child maltreatment [45]. Furthermore, the drop-out of children absent from school the day the questionnaire was given out may distort the results since this might be a group of more disadvantaged pupils. Their absence could depend on truancy or illness and lead to an underestimation of the true prevalence of physical abuse as well as poor health.

One strength of the present study implies the use of anonymous questionnaire in the school situation (away from home and without parental immediate influence) provides an opportunity to obtain information about the real extent of children’s exposure to and the implications of CPA. The confidentiality of the survey makes it possible for children to answer these sensitive issues. Only eight percent of the abused had told any authority about their exposure implying that this study-design captures a quite different spectrum of children than a clinical sample would do. The fact that the study was performed in a representative group of the general youth population, in other words a non-clinical group of children, is another strength of this study, not to mention the large sample and the overall high response rate which have made it possible to perform analyses of different subgroups. The addition of the question of last year exposure, is an improvement compared with many previous studies since it allows for more accurate reporting of prevalence avoiding recall bias to the same extent that occurs if one asks for life-time experiences [20]. However, it is still important to measure both lifetime and point prevalence (last year exposure) to examine the associations between abuse and poor health.

Finally, it is important to note that in this study analyses were performed in a group consisting only of exposed children and characteristics of CPA and other background factors are solely compared to each other. In studies also including non-exposed children, figures of correlations between abuse and poor health would probably been higher since previous studies show that exposed children report poor health more than not exposed children do [18, 19].

This study adds to the literature, suggestions of how different characteristics and background factors are associated with poor health among children exposed to CPA. In the existing literature on child maltreatment there are many studies on how single characteristics of the abuse are associated with outcomes of poor health. To our best knowledge, no previous studies have compared these factors in order to examine which ones that have the greatest impact on health or how they interact with each other.

## Conclusion

Despite the limitations that cross-sectional studies imply, this study provides new knowledge about factors associated with poor health among physically abused children. It describes details of CPA that have significant associations to different aspects of poor health and thus what needs to be addressed by professionals within mental health providers and social services. Understanding how different factors contribute to different health outcomes for exposed children is important in future research and needs further studies.

## Abbreviations

CPA: Child physical abuse
CTS: Conflict Tactic Scale
OR: odds ratio
